# Transcriptome Analyses of Pre-parasitic and Parasitic *Meloidogyne Chitwoodi* Race 1 to Identify Putative Effector Genes

**DOI:** 10.21307/jofnem-2021-084

**Published:** 2021-10-12

**Authors:** Lei Zhang, Cynthia Gleason

**Affiliations:** 1 Plant Pathology Department, Washington State University, Pullman, WA 9916; 2 Department of Botany and Plant Pathology, Purdue University, West Lafayette, IN 47907; 3 Department of Entomology, Purdue University, West Lafayette, IN 47907

**Keywords:** Differentially expressed genes, Host-parasitic relationship, Meloidogyne, Molecular biology, Transcriptome

## Abstract

*Meloidogyne chitwoodi* is a root-knot nematode that is a major pest of potato in the northwestern United States. Due to the lack of resistance against root-knot nematodes in potato, research has been undertaken to understand the *M. chitwoodi*-potato interaction at the molecular level. To identify the nematode genes that are playing roles in parasitism, we have performed transcriptome analyses on pre-parasitic and parasitic *M. chitwoodi* juveniles in susceptible potato. We compared gene expression profiles and identified genes that were significantly up- or down-regulated during nematode parasitism. Because parasitism proteins are typically secreted by the nematode to facilitate infection of host roots, we focused on the genes that encoded proteins that were predicted to be secreted. We found that approximately 34% (43/127) of the genes in the predicted secretome encoded proteins with no significant homology in the public genome databases, and 12% (15/127) encoded either a known effector, putative effectors or putative esophageal gland cell proteins. The transcriptome analyses of *M. chitwoodi* at the pre-parasitic and parasitic life stages shed light on the genes involved in nematode parasitism.

Root-knot nematodes are microscopic, endoparasitic roundworms that are a major limiting factor in the production of potatoes, which are the fourth most important food crop in the world (Birch et al., 2012; Lima et al., 2018). *Meloidogyne chitwoodi* (Golden et al., 1980) (Tylenchida: Meloidogynidae) is a root-knot nematode with limited worldwide distribution, and in the United States, *M. chitwoodi* is widely spread in the three states (Washington, Oregon, and Idaho) that produce over half of US potatoes (CABI/EPPO, 2012; Zasada et al., 2018).

*M. chitwoodi* second stage juveniles (J2s) hatch from the eggs and invade roots behind the root tip. They migrate intercellularly towards the differentiating vascular cylinder. In the case of tubers, the J2 will enter through wounds or lenticels. The nematode chooses 4 to 8 plant cells as feeding sites, called giant cells, which serve as the only source of nematode nutrition (Bird, 1961; Finley, 1981). Once feeding, the J2s become sedentary and molt three times into the adult life stage, and the females will lay eggs. Meanwhile, hyperplasia of surrounding root cells results in galling, which is a classic symptom of root-knot nematode infection. *M. chitwoodi* infected tubers develop galls on the skin. In addition to the galling, small dark spots appear in the tuber flesh surrounding the adult females. Tubers with 6 or more infection sites are regarded as culls (Pinkerton et al., 1986), and if as few as 5% to 15% of the tubers display these visual defects, the entire crop will be devalued or rejected (Ingham et al., 2000; Ingham et al., 2007). Because there are no commercially available potato cultivars resistant to root-knot nematodes, conventional potato production relies heavily on chemical controls to manage nematodes (Ingham et al., 2000; King and Taberna, 2013). Although nematicides are effective against root-knot nematodes, there are concerns about the potential negative effects of these chemicals on human health and the environment (Porter et al., 2009). In order to reduce our reliance on nematicides and develop new forms of root-knot nematode resistance in potato, we must better our understanding of the plant-nematode interaction at the molecular level.

Because of their critical roles in nematode parasitism, there has been a lot of interest in identifying root-knot nematode genes that are upregulated in expression during their parasitic life stages (Barcala et al., 2010; Caillaud et al., 2008; Dubreuil et al., 2007; Lee et al., 2019; Shukla et al., 2018). The nematode genes with roles in parasitism are referred to as ‘effector genes.’ Typically, effector genes encode secreted proteins with roles in host defense suppression and/or in feeding site formation (Vieira and Gleason, 2019). One of the first steps in identifying effectors in root-knot nematodes is to identify genes that are expressed during the parasitic life stages. Transcriptome analysis of sedentary endoparasitic nematodes requires either the removal of the nematodes from the plant tissue prior to RNA sequencing (Kooliyottil et al., 2019) or dual RNA-sequencing (RNA-seq), in which the host roots and the invading nematodes are simultaneously sequenced (Westermann et al., 2012). Recently a dual RNA-seq project was undertaken on *M. graminicola-*infected rice (Petitot et al., 2016). The gene expression profiling was followed by a secreted protein prediction pipeline to identify putative *M. graminicola* effectors (Petitot et al., 2016). The dual RNA-seq approach has also been used to identify effectors from the cyst nematode *Heterodera schachtii* (Elashry et al., 2020), further emphasizing that dual RNA-sequencing is a useful tool in plant parasitic nematode effector gene discovery.

A recent publication looked at the expression of potato genes during *M. chitwoodi* race 1 infection (Bali et al., 2019). Here, our goal was to identify *M. chitwoodi* genes differentially expressed during potato parasitism by using dual RNA-seq. The list of identified genes was further narrowed by using bioinformatic tools to identify genes with putative secretion signals and no predicted transmembrane domains. These criteria are indicative of nematode genes that encode secreted proteins; this study lays the groundwork for the future identification and characterization of *M. chitwoodi* secreted effectors.

## Materials and Methods

### Nematode cultures and inoculation of potato plants

**Figure S1:**
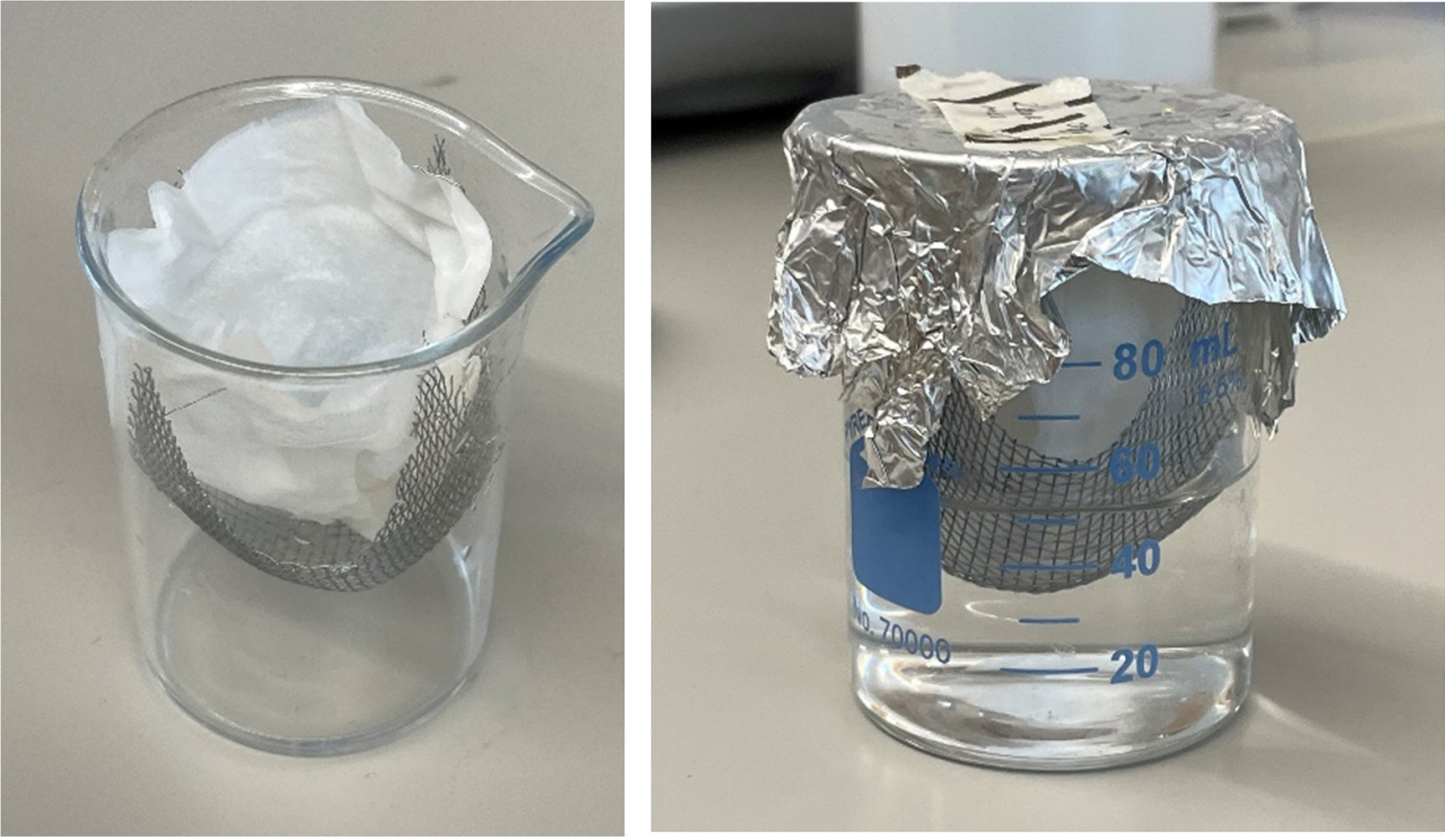
Modified Baermann funnel apparatus diagram. The modified Baermann funnel apparatus uses a 100 mL beaker instead of the traditional funnel. Mesh is placed into the mouth of the beaker and up to 4 layers of Kimwipes are placed on the mesh. Nematode eggs (or soil) are placed onto the paper and immersed in water. After three days, the paper and mesh are removed and the nematodes sitting at the bottom of the beaker are collected. The modified apparatus allows for J2 collection from small volumes of soil or isolated eggs.

The *Meloidogyne chitwoodi* isolate race 1 egg inoculum was initially provided by Dr. Charles Brown (USDA-ARS), and *M. chitwoodi* race 1 was maintained on the susceptible tomato *Solanum lycopersicum* cv. Rutgers under greenhouse conditions. To obtain nematode eggs from inoculated tomato plants, tomato roots were cut into small pieces and rinsed in a 0.6% sodium hypochlorite solution for 3 min. The solution was then passed through a set of sieves (pore size of 125, 45, 25 μm). Nematode eggs were collected on the 25 μm sieve and then further purified using sucrose floatation (Jacob and van Bezooijen, 1984). The eggs were suspended in 0.1% Plant Preservative Mixture (Plant Cell Technology) and incubated in a modified Baermann beaker (Figure S1) in darkness at room temperature. Hatched infective J2s were collected after 3 days of incubation.

For inoculation of potato plants with *M. chitwoodi*, three-week-old tissue culture Russet Burbank plantlets were transferred to 500-mL cone-tainers filled with sand and grown in growth chambers (14-hr light/10-hr darkness, 23^o^C) for 14 days. The Russet Burbank plants were then inoculated with 500 freshly hatched *M. chitwoodi* race 1 J2s. Root samples were harvested at 4-, 8- and 16-day-post-inoculation (dpi) and were stained with acid fuchsin solution (0.35% acid fuchsin, 25% acetic acid) to visualize nematodes within roots (Bybd et al., 1983) on a Nikkon Eclipse Ti inverted microscope at × 40 magnification.

### Sample preparation for RNA-seq experiment

For RNA-seq experiment, newly hatched *M. chitwoodi* race 1 J2s and galled potato root tissues at 8 dpi were collected, respectively. Three biological samples of *M. chitwoodi* race 1 J2s were also collected. Total nematode RNA was extracted using PureLink™ RNA Mini Kit (ThermoFisher). Following the inoculation of potato plants with 500 *M. chitwoodi* J2s (procedure described above), Russet Burbank roots were harvested at 8 dpi, and the infected roots were checked under a stereomicroscope (Zeiss zoom stereomicrope) to collect galled root tissues where nematodes resided. Galled root tissues from two individual potato plants were combined as one biological replicate, and three biological replicates were collected for 8 dpi samples. Total RNA was extracted from root samples using RNeasy Plant Mini Kit (QIAGEN, Germany). High quality total RNA (1-2 µg, 260/280 = 1.8-2.2, with RIN value of RNA of at least 8.5) was packed on dry ice and sent to Novogene Co. Ltd for library preparation and sequencing (30M reads per sample with PE150) using the Illumina platform.

### RNA-seq data analysis

The raw sequencing data were trimmed using Trimmomatic-v0.38 to remove adapters and low-quality sequences (Bolger et al., 2014). The quality of trimmed sequences was checked using FastQC-v0.11.7 (http://www.bioinformatics.babraham.ac.uk/projects/fastqc). The trimmed sequences were evaluated according to the summary output of FastQC, and threshold of per base sequence quality was set as 20 by default. The trimmed data was analyzed using Tophat/2.1.1 to map to the *M. chitwoodi* race 1 draft genome (Bali et al., 2021). Mapped read counts were calculated using Cuffdiff of the Cufflinksv2.2.1 package, and the gene read counts were converted to tab-delimited text file (Trapnell et al., 2012). The differentially expressed genes (DEGs, adjusted p-value < 0.05) between *M. chitwoodi* J2s and at 8 dpi (n = 3 biological samples for each time point) were analyzed using the Cuffdiff.

### Prediction of secreted proteins

We predicted secreted proteins from the *M. chitwoodi* race 1 genes up-regulated at 8 dpi based on the criteria: presences of N-terminus signal peptide and absence of transmembrane domains. First, the coding DNA sequences (CDS) of *M. chitwoodi* race 1 genes were predicted using the TransDecoder v5.1.0 with minimal length of deduced proteins set as 30 amino acids (Haas et al., 2013). The deduced protein sequences of up-regulated genes were used to predict presence of N-terminus signal peptide for secretion using the software SignalP version 5.0 (Almagro Armenteros et al., 2019; Nielsen, 2017). Proteins predicted to have Signal Peptide (Sec/SPI) likelihood above 0.6 were retained for further analyses. These protein sequences were then subjected to TMHMM v. 2.0 analysis, which predicts membrane proteins (Krogh et al., 2001). Proteins were classified as secreted proteins if they either had no predicted transmembrane domains or only had predicted transmembrane domains in the secretion signal sequence.

### Real time PCR validation

For gene expression analysis, potato galled root tissues inoculated with 500 *M. chitwoodi* race 1 J2s were collected at 4-, 8- and 16-day-post-inoculation. Simultaneously, freshly hatched *M. chitwoodi* race 1 pre-parasitic J2s were also collected. Total RNA was extracted from galled root tissues and J2s separately and was treated with DNA-free™ DNA Removal Kit (ThermoFisher) to remove residual DNA. cDNA was synthesized using ProtoScript II First Strand cDNA Synthesis Kit (New England Biolabs, USA) with oligo-dT primer. qRT-PCR was performed using SsoAdvanced™ Universal SYBR^®^ Green Supermix on a CFX96 Real-Time PCR Detection System (Bio-Rad, USA). The qRT-PCR conditions were: 95^o^C for 3 min, 40 cycles of 95^o^C for 15 sec, 53^o^C for 15 sec and 72^o^C for 20 sec, and followed by a melting curve analysis from 65^o^C to 95^o^C with 0.5^o^C increased incrementally at 5 sec increments. Expression levels of genes of interest in nematode were normalized to the expression of *M. chitwoodi* housekeeping gene Internal transcribed spacer 2 (ITS2) rRNA. The relative expression levels of each target gene at 4-, 8- or 16-dpi were calculated by comparing with those in *M. chitwoodi* race 1 J2s using the 2–ΔΔCt method (Livak and Schmittgen, 2001). The primer pairs used for RT-qPCR are summarized in Table 1. Each qRT-PCR experiment consisted of two technical replicates per time-course of each gene, and the average of three biological replicates. Statistical tests were performed using GraphPad Prism version 8.00 for Windows (GraphPad Software, La Jolla California USA, www.graphpad.com).

**Table 1.** Primers for qRT-PCR.

Name	5′-3′
McITS2 forward	GGGGTCAAACCCTTTGGCACGTCTGG
McITS2 reverse	GCGGGTGATCTCGACTGAGTTCAGG
Mc00773 forward	AAGTTGCCGATAGCATTGCG
Mc00773 reverse	TCCGGAAGAGCATGACGAAT
Mc03439 forward	TGCAGTTGCTGAGAGTTGTC
Mc03439 reverse	TGCATGGTGGAATAACCAAAGT
Mc03992 forward	TGATCGTTCGACTTCTGGACA
Mc03992 reverse	GCACGAGTGCCTTGAACTTG
Mc04677 forward	CTGCACATCAGAAGAAAAATGCT
Mc04677 reverse	TCCACAGGGTCACAACTTCC
Mc04921 forward	TCGTGTTAGCGGTGATGGTT
Mc04921 reverse	CGTTTCGGTGCCAAGTTCAG
Mc08895 forward	TCTTTGCACGAAGTTGATCG
Mc08895 reverse	TTTAATCCATTTACAAAAAGCCCT
Mc10400 forward	CAAGGAGGTGGAAAAACGCC
Mc10400 reverse	GGGTTCTTGATTCCCAACCG
Mc01565 forward	CAGCTGAATCTTCTGCCCCA
Mc01565 reverse	AATAGCAACGGCTGGAGCTT
Mc06869 forward	CCACATCATCATCATCATTCAAGT
Mc06869 reverse	CCCATGGCCAAGTTGAACC
Mc08299 forward	GAATTACGCCGCTTTCTTGGT
Mc08299 reverse	TGCACATTCAGGCCACTCAT
Mc07118 forward	TGGTTGTTATGAACATTCTGGCG
Mc07118 reverse	CCAGGTTTGTTAGGTAAGGCAG
Mc10102 forward	TCCACATCATCCTGGAGGTC
Mc10102 reverse	ATGAAAAGCACCAGCCCCAT

## Results

### Differential gene expression of Meloidogyne chitwoodi during early infection stage in potato

**Figure 1:**
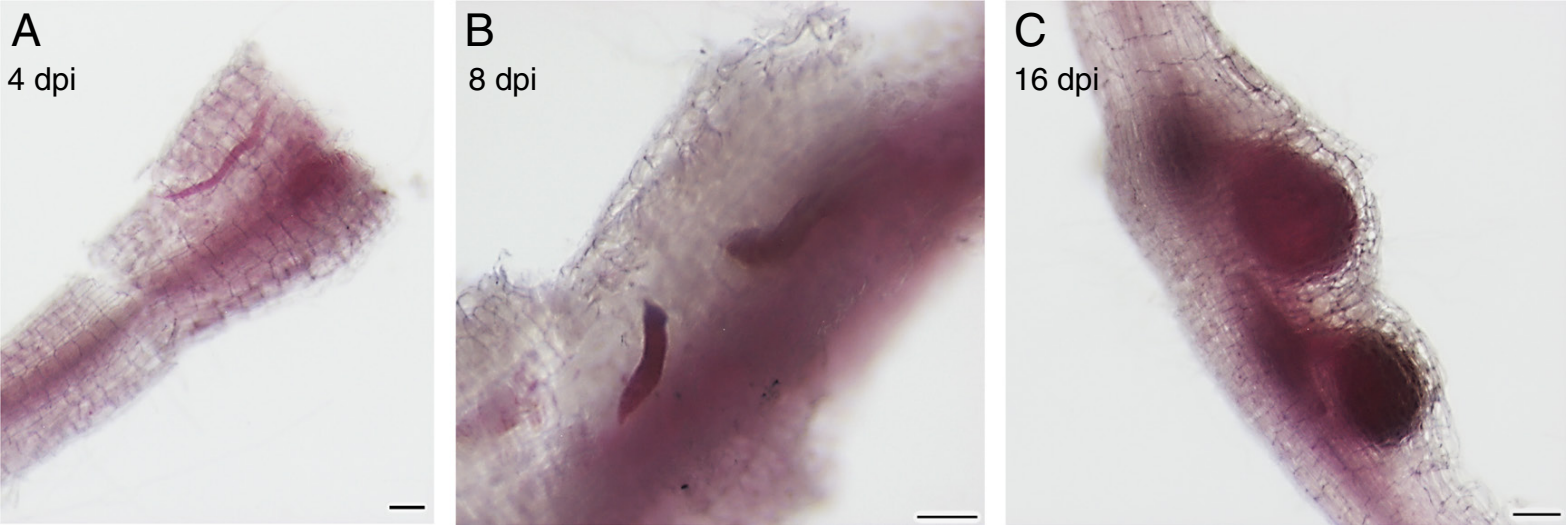
Acid fuchsin staining of *Meloidogyne chitwoodi* in potato roots at 4 (A), 8 (B) and 16 (C) days post inoculation (dpi). Bars, 50 µm.

Root-knot nematode effector genes critical for parasitism are typically up-regulated during early infection stages, when the nematodes enter the root, evade plant defenses, and form their giant cells. To identify *M. chitwoodi* genes expressed during early infection stages, we first established infection timepoints in our experimental system. We monitored nematode infections at 4-, 8- and 16-days post-inoculation (dpi) by staining the roots with acid fuchsin, allowing us to observe nematodes within the roots. At 4 dpi, J2 could be seen within the roots, indicating that root penetration had occurred. At 8 dpi, most nematodes within the roots were swollen, but still in the J2 stage. The swollen bodies indicated that the nematodes had begun to feed but had not yet molted to the next life stage. At 16 dpi, galls on the roots were clearly visible and the nematodes were in the globose J4 or young female stage (Fig. 1). Based on our observations and experimental setup, at 4 dpi, the juveniles were still migrating through the roots, and by 8 dpi, the nematodes had settled and started to feed. Because we wanted to capture the J2 stage when initial feeding and giant cell formation occurs, we chose 8 dpi as the timepoint for the RNA-seq experiment.

Our next goal was to compare gene expression profiles between pre-parasitic and parasitic J2 to determine which nematode genes are differentially regulated during parasitism. We collected freshly hatched *M. chitwoodi* Race 1 J2 as the pre-parasitic J2 samples. To obtain the parasitic nematodes, Russet Burbank potato plants were inoculated with *M. chitwoodi* Race 1 J2 and galled root tissues were collected at 8 dpi. We collected whole galls to avoid isolating nematodes from the roots, which meant we had a mixture of plant and nematode tissue in the sample. Six cDNA libraries were constructed – three libraries of pre-parasitic J2 and three libraries of galls isolated at 8 dpi. The libraries were sequenced using an Illumina platform for paired end 150-bp reads, generating between 43 and 55 million raw read pairs for each library. The RNA-seq raw reads were pre-processed to remove low-quality bases and adapter sequences, resulting in between 40 and 52 million clean read pairs (Table 2). Interestingly, the GC content of individual libraries were different among samples: an average of 34% GC content for the *M. chitwoodi* J2 samples and 41% of GC content for the 8 dpi samples. The low GC content of the *M. chitwoodi* J2 transcripts corresponds to the low GC content of the *M. chitwoodi* and other root-knot nematode genomes (Koutsovoulos et al., 2020; Paganini et al., 2012). The higher GC content of the 8 dpi samples may be due to the mixture of plant and nematode reads at 8 dpi.

**Table 2.** *Meloidogyne chitwoodi* RNA-seq libraries and mapping to genome.

Library	Bio-replicate	Read pairs	Read pairs mapped to genome	Mapping rate (%)
Pre-parasitic J2	A	51 950 815	25 570 659	49.60%
Pre-parasitic J2	B	43 883 143	21 477 892	49.00%
Pre-parasitic J2	C	44 867 529	22 072 545	49.20%
Mc1-potato 8 dpi	A	46 115 017	1 572 404	3.40%
Mc1-potato 8 dpi	B	40 726 172	1 294 772	3.20%
Mc1-potato 8 dpi	C	40 068 646	1 357 759	3.40%

**Figure 2:**
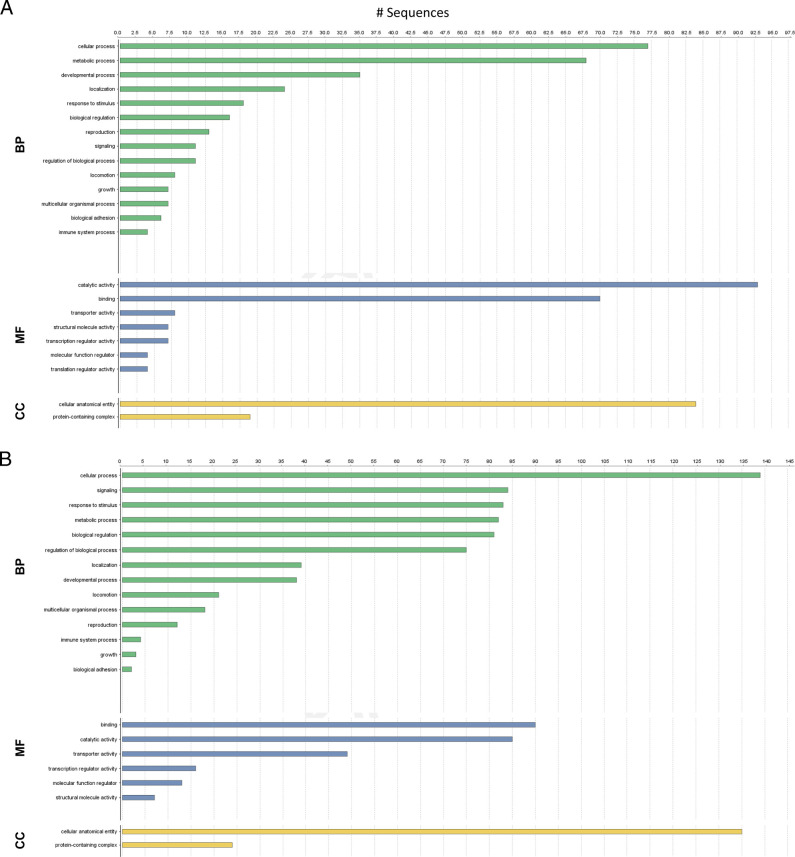
Gene Ontology (GO) term level 2 categories of genes for up (A) and down (B) regulated genes from *M. chitwoodi* parasitic stage compared to pre-parasitic J2. Bars show the number of sequences with annotations under each term. BP = biological processes, MP = molecular processes, CC = cellular function.

The differentially expressed genes (DEGs) of *M. chitwoodi* between J2 and 8 dpi were analyzed using the TopHat and Cufflinks/Cuffdiff pipeline (Trapnell et al., 2012). First, the RNA-seq read pairs were mapped to the *M. chitwoodi* race 1 genome using TopHat2. Mapping the reads from the J2 libraries generated 21 to 25 million mapped read pairs. Meanwhile, mapping reads from the 8dpi gall library resulted in 1.2 to 1.5 million mapped read pairs. By mapping to the *M. chitwoodi* race 1 genome, we removed the plant sequences from our dual RNA-seq experiment (Table 2). The reduction in the read pairs suggest that the majority of the reads from the dual sequencing were from potato. Next, we used Cufflinks to merge the information from all six libraries to assemble a *M. chitwoodi* transcriptome. DEGs between pre-parasitic and parasitic (8 dpi) J2 were determined by Cuffdiff. In the parasitic nematodes, we found 693 *M. chitwoodi* genes up-regulated (fold-change ≥ 4 and adjusted *P* < 0.05) and 1727 *M. chitwoodi* genes down-regulated (at least a 4-fold decrease and adjusted *P* < 0.05) (Supplementary Table 1, https://figshare.com/articles/dataset/Zhang_and_Gleason/16746286). To limit the number of genes in our subsequent analyses, we focused on genes encoding proteins of 30 or more amino acids. This left us with 679 genes up-regulated and 1570 genes down-regulated. A BLASTx-fast search of the NCBI non-redundant (nr) database found that of the 679 upregulated genes, 341 (50%) did not have any Blast hits (E value ≤ 1.0E-3) (Supplementary Table 2, https://figshare.com/articles/dataset/Zhang_and_Gleason/16746286). Second level GO term analysis of the up-regulated genes indicate that sequences related to cellular, metabolic, and developmental processes were enriched during nematode parasitism (Fig. 2A). Of the 1570 down regulated genes, 408 (26%) did not have any Blast hits (E value ≤ 1.0E-3) (Supplementary Table 3, https://figshare.com/articles/dataset/Zhang_and_Gleason/16746286). Second level GO term analysis of the down-regulated genes indicate that sequences related to cellular, signaling, and response to stimulus were enriched in pre-parasitic nematodes (Fig. 2B). When comparing genes expressed in the pre-parasitic J2 versus genes expressed in the parasitic nematode, we comprised a list of the top 20 most differentially expressed genes and their annotations from the BLASTx homology search (Table 3).

**Table 3.** Top 10 most differentially expressed genes in parasitic *M. chitwoodi juveniles.*

		Log FC	Description
Up-regulated	1	12.2878	Putative cuticular collagen
	2	10.8863	---NA---
	3	10.6047	Putative esophageal gland cell secretory protein 14
	4	10.5679	---NA---
	5	10.5294	Hydroxyacyl-coenzyme A dehydrogenase, mitochondrial
	6	9.2692	---NA---
	7	9.21531	---NA---
	8	9.21423	Nematode cuticle collagen domain protein
	9	9.1679	Saposin-like type B, 1 domain and Saposin B domain and Saposin-like domain-containing protein
	10	9.0144	---NA---
Down-regulated	1	-11.012	C-type lectin domain-containing protein
	2	-10.711	Mucin-like protein-1
	3	-10.681	SCP domain-containing protein
	4	-10.443	---NA---
	5	-10.308	Beta-1,4-endoglucanase
	6	-10.234	C-type lectin domain-containing protein
	7	-10.205	Mucin-like protein-1
	8	-9.9796	---NA---
	9	-9.975	---NA---
	10	-9.8201	Hypothetical protein Mgra_00005110, partial

### Prediction of secreted proteins among *M. chitwoodi* genes up-regulated at early infection stage

**Figure 3:**
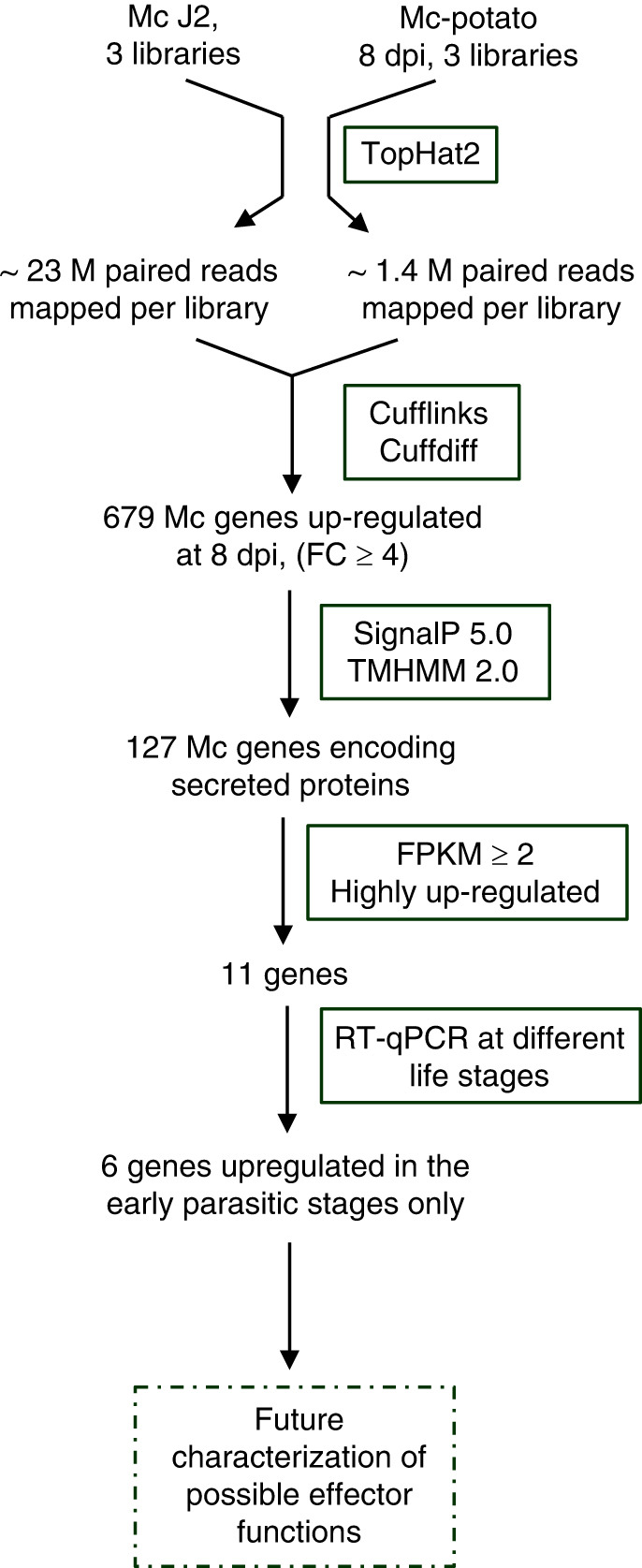
Flowchart summary of RNA-seq analysis and prediction of *Meloidogyne chitwoodi* genes involved in parasitism. Bioinformatic and other tools are boxed. FC, fold change. FPKM, fragments per kilobase of transcript per million mapped reads.

We hypothesized that nematode genes induced at early infection stages may be important for successful infection of host plants, therefore, we focused on the 679 genes that were significantly up-regulated at 8 dpi (Fig. 3, Supplementary Table 2, https://figshare.com/articles/dataset/Zhang_and_Gleason/16746286). We performed a bioinformatic analysis on the 679 *M. chitwoodi* genes up-regulated at 8 dpi to predict if the genes encode secreted proteins. The prediction of secretion was based on two criteria: the presence of a signal peptide at the N-terminus of the peptide sequence and the lack of any transmembrane domains. The SignalP 5 program predicted that 133 of the proteins contained a signal peptide at the N-terminus. Among these 133 sequences, 6 were excluded from further analysis because the TMHMM2 predictions indicated they contained one or more transmembrane domains within the protein sequence, minus the signal peptide sequence. Therefore, *in silico* analyses identified 127 *M. chitwoodi* genes (18.7% of the 679 up-regulated genes) encoding predicted secreted proteins (Supplemental Table 4, https://figshare.com/articles/dataset/Zhang_and_Gleason/16746286).

**Table 4.** Information about the 11 *M. chitwoodi* genes analyzed by qRT-PCR at different life stages.

Gene ID (aa)	BLASTx nr	BLASTx % query cover	BLASTx e-value	Predicted domains	FPKM J2	FPKM 8 dpi
Mc07118 (171)	*Meloidogyne graminicola* MSP18 mRNA, complete cds, Genbank: MK628546.1	84	7.00E-40	N/A	2	73.9
Mc10102 (103)	Unnamed protein product [*Meloidogyne enterolobii*] Genbank: CAD2180454.1	79	4.00E-46	Cytochrome b5, heme binding domain	16.7	418.6
Mc08299 (207)	Cathepsin B [*Meloidogyne graminicola*]Genbank KAF7630769.1	89	7.00E-45	cysteine proteinase	26.4	476
Mc04921 (544)	hypothetical protein Mgra_00005223 [*Meloidogyne graminicola*] Genbank KAF7635403.1	34	6.00E-74	Thioredoxin-like fold	2.9	50.4
Mc06869 (63)	N/A			N/A	39.2	1704.9
Mc03992 (306)	Hypothetical protein Mgra_00005223 [*Meloidogyne graminicola*] Genbank KAF76334147.1	49	3.20E-02	N/A	4.3	69.8
Mc01565 (430)	Unnamed protein product [*Meloidogyne enterolobii*] Genbank: CAD2124200.1	18	2.00E-22	N/A	7.3	288.1
Mc08895 (332)	Unnamed protein product [*Meloidogyne enterolobii*] Genbank: CAD2174631.1	32	5.00E-25	N/A	3.5	123.7
Mc04677 (315)	Hypothetical protein Mgra_00005223 [*Meloidogyne graminicola*] Genbank KAF7635986.1	59	7.00E-54	N/A	2.3	51.8
Mc03439 (79)	Hypothetical protein Mgra_00005223 [*Meloidogyne graminicola*] Genbank KAF7630992.1	97	1.00E-23	N/A	25.8	1319.5
Mc10400 (130)	N/A			N/A	7.2	154.8

Using the sequence information for the 127 identified putative secreted proteins, we performed a Blastx search of the NCBI nr database. We found that 62.9% (80/127) did not have any BLAST hits (E value ≤ 1.0E-3) (Supplemental Table 4, https://figshare.com/articles/dataset/Zhang_and_Gleason/16746286). Twelve of the genes (12/127) were annotated as “putative esophageal gland cell secretory protein” (Huang et al., 2003). Three known effectors were found in the annotations of the 127 *M. chitwoodi* genes, including VAP1, which was originally discovered in cyst nematodes as an effector that suppresses basal plant defense responses (Lozano-Torres et al., 2014). A gene encoding a chorismate mutase domain-containing protein was also identified in our in-silico *M. chitwoodi* secretome analysis. Root-knot nematode chorismate mutases may be altering salicylic acid levels within the host cells to suppress plant immune responses (Huang et al., 2005; Lambert et al., 1999; Wang et al., 2018). The third known effector found in our list of *M. chitwoodi* genes is homologous to the candidate secreted effector Minc11888, which was previously identified in *M. incognita* and is highly expressed in the parasitic juvenile stages (Nguyen et al., 2018).

Similarly, to assess protein homology, a BLASTp analysis of the 127 proteins using the nr database was performed. The analysis revealed that 66% (84/127) had significant hits (60% query coverage and E value ≤ 1.0E-3), to plant parasitic nematodes (Supplemental Table 5, https://figshare.com/articles/dataset/Zhang_and_Gleason/16746286). Interestingly, 53.5% (45/84) of those genes were hypothetical or unnamed proteins from the rice root-knot nematode (*M. graminicola*), which is a close relative to *M. chitwoodi* in the Meloidogynidae (Bert et al., 2008; Phan et al., 2020). We also found that 35/84 genes in our BLASTp analysis had top hits to genes encoding unnamed proteins in *M. enterolobii*, perhaps owing to the fact that *M. enterolobii* has one of the most complete genomes available for root-knot nematodes at this time (Koutsovoulos et al., 2020).

### Real-time PCR validation on highly up-regulated genes

**Figure 4:**
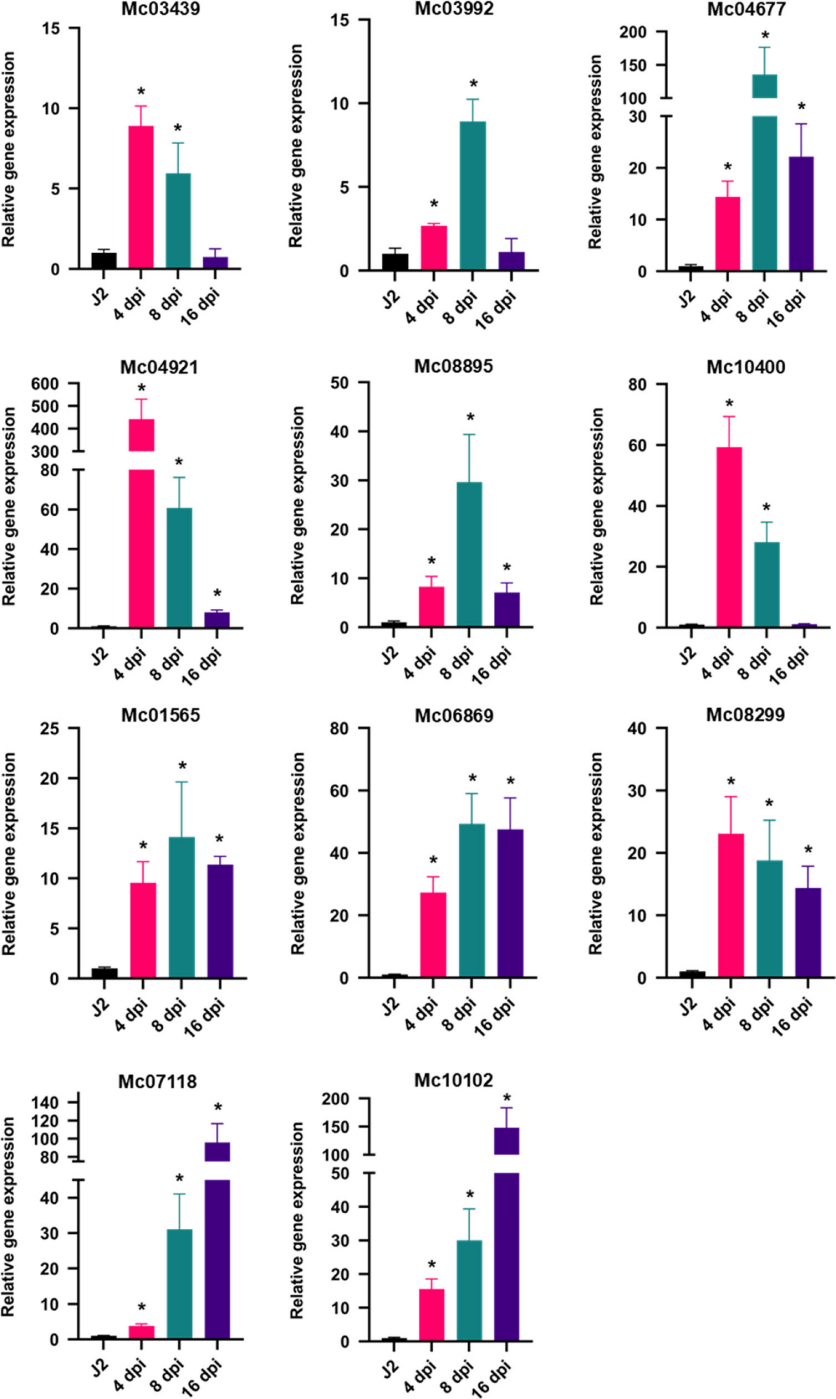
The expression level of selected *M. chitwoodi* genes in J2 and in parasitic nematodes in potato roots at 4, 8, and 16 dpi. Mc*ITS2* was used as the reference gene. The results show the fold change relative the juvenile stage, n = 3 ± SEM, * *P* < 0.05 using two-tailed Student’s *t* test.

To validate our list of up-regulated genes, we selected 11 genes from the list of 127 that were highly up-regulated at 8 dpi (FC > 10 and FPKM ≥ 2, Table 4). We measured the expression of these 11 genes by quantitative reverse transcription polymerase chain reaction (RT-qPCR) in different nematode life stages: pre-parasitic J2 and parasitic nematodes in potato roots at 4, 8, and 16 dpi. Expression of 6 genes (*Mc03439*, *Mc03992*, *Mc04677*, *Mc04921*, *Mc08895* and *Mc10400*) peaked at the early infection stages (4 or 8 dpi) and decreased at a later life stage (16 dpi) (Fig. 4). For the other genes, the expression levels were either similar across all infection stages (*Mc01565*, *Mc06869*, *Mc08299*) or highly expressed at the later infection stage, 16 dpi (*Mc07118* and *Mc10102*) (Fig. 4). All 11 genes were confirmed to be expressed at higher levels at 8 dpi by RT-qPCR compared to the J2 stage, confirming our transcriptome data (Fig. 4). These results highlight the fidelity of dual RNA-seq analyses to identify differentially expressing genes.

## Discussion

Nematode success depends on the production and secretion of effectors. Therefore, effector identification is critical for understanding the compatible interaction with the host. The first step in effector identification is the discovery of differentially expressed genes during the nematode parasitic life stages. In a previous report, effector genes of *M. chitwoodi* were mined from an EST library constructed from *M. chitwoodi* eggs, J2s and females, and over three hundred predicted secreted effectors were identified (Roze et al., 2008). This study did not analyze gene expression specifically at the early parasitic life stages of the nematode, and as a result, the large list of genes in the secretome may include genes not related to parasitism. Because root-knot nematodes are not transformable, it would be a very labor-intensive task to try to functionally characterize over three hundred nematode genes. In this study we have compared the transcriptomic datasets between pre-parasitic and parasitic *M. chitwoodi* juveniles and generated the first comprehensive analysis of *M. chitwoodi* genes that are expressed during parasitism and that encode predicted secreted proteins. We have narrowed our list to 127 genes, of which the majority encode pioneer proteins. We focused on upregulated genes because we are interested in nematode effectors, which are secreted during infection to promote parasitism through the suppression of the host defense responses and/or the creation of the feeding site (Vieira and Gleason, 2019). Although the list of upregulated genes encoding secreted proteins is still relatively large, future experiments that would narrow this list further include performing additional gene expression analyses over a time course or finding transcripts that localize to the nematode secretory organs through in situ hybridization experiments.
